# Accessing new 2D semiconductors with optical band gap: synthesis of iron-intercalated titanium diselenide thin films *via* LPCVD†

**DOI:** 10.1039/c8ra03174f

**Published:** 2018-06-20

**Authors:** Clara Sanchez-Perez, Caroline E. Knapp, Ross H. Colman, Carlos Sotelo-Vazquez, Raija Oilunkaniemi, Risto S. Laitinen, Claire J. Carmalt

**Affiliations:** University College London, Department of Chemistry 20 Gordon St London WC1H 0AJ UK c.j.carmalt@ucl.ac.uk; Laboratory of Inorganic Chemistry, Environmental and Chemical Engineering, University of Oulu P. O. Box 3000 FI-90014 Oulu Finland risto.laitinen@oulu.fi; Charles University, Faculty of Mathematics and Physics, Department of Condensed Matter Physics Ke Karlovu 3 Prague 2 Czech Republic

## Abstract

Fe-doped TiSe_2_ thin-films were synthesized *via* low pressure chemical vapor deposition (LPCVD) of a single source precursor: [Fe(η^5^-C_5_H_4_Se)_2_Ti(η^5^-C_5_H_5_)_2_]_2_ (1). Samples were heated at 1000 °C for 1–18 h and cooled to room temperature following two different protocols, which promoted the formation of different phases. The resulting films were analyzed by grazing incidence X-ray diffraction (GIXRD), X-ray photoelectron spectroscopy (XPS), scanning electron microscope (SEM) and UV/vis spectroscopy. An investigation of the Fe doping limit from a parallel pyrolysis study of Fe_*x*_TiSe_2_ powders produced *in situ* during LPCVD depositions has shown an increase in the Fe–TiSe_2_–Fe layer width with Fe at% increase. Powders were analyzed using powder X-ray diffraction (PXRD) involving Rietveld refinement and XPS. UV/vis measurements of the semiconducting thin films show a shift in band gap with iron doping from 0.1 eV (TiSe_2_) to 1.46 eV (Fe_0.46_TiSe_2_).

## Abstract 2

Fe-doped TiSe_2_ thin-films were synthesized *via* low pressure chemical vapor deposition (LPCVD) of a single source precursor: [Fe(η^5^-C_5_H_4_Se)_2_Ti(η^5^-C_5_H_5_)_2_]_2_ (1).

## Graphical abstract

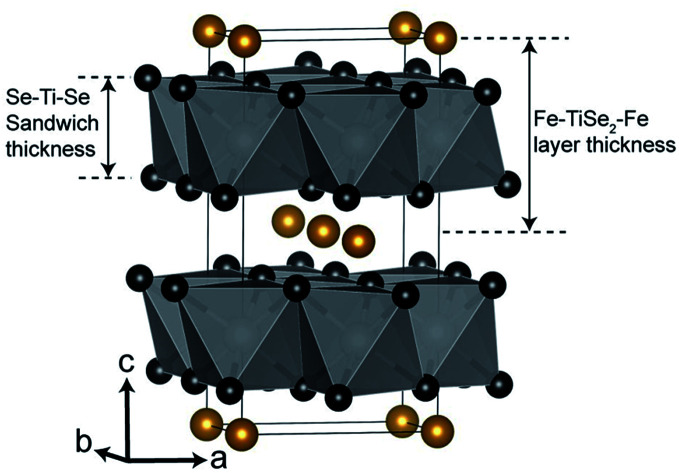

## Introduction

The continuous search for high-speed/low-power electronics beyond the current silicon-based devices has incentivised the research on these layered materials with band gaps, redirecting interest from graphene to 2D group 4 and 6 TMDs materials and heterostructures with tuneable band alignments for a variety of nanoelectronic/optoelectronic applications.^1–5^ The design of high-efficiency materials which can convert solar to electrical energy is a continuously increasing research topic, in which the main path nowadays involves thin-film technology.^6^ Early transition metal chalcogenides have been reported to have applications as cathode materials for rechargeable batteries.^7^ Many of them are semiconductors with band gaps lying within the UV-visible region, and are predicted to be strong absorbers of light, which makes them interesting candidates in the search of next generation solar cell energy devices.^8^

Group 4 transition metal diselenides (TMDs) crystallize in the 1T-CdI_2_ structure *P*3̄*m*1, with a hexagonal layer of transition metal sandwiched between two hexagonal layers of chalcogen atoms (S, Se, Te). In the crystal structure, layers stack along the *c* axis *via* van der Waals forces.^9^ Many kinds of atoms and organic molecules can be intercalated into the van der Waals gap sites of layered TMDs, causing dramatic changes in physical properties of the host materials^10,11^ and generating a wide variety of magnetic orderings in these so-called intercalated transition metal dichalcogenides (ITMDs).^10^ Owing to their ability to exist in more than one oxidation state, investigation of transition metals and their role in electronic and transport properties of the aforementioned complexes is of great interest.^12^ Ternary chalcogenides of the series Fe_*x*_TiSe_2_ have a defect NiAs-type lattice.^13,14^ The anions are arranged in hexagonal closed-pack layers sandwiching octahedrally coordinated Ti^4+^ cations. Previous studies of the Fe_*x*_TiSe_2_ system as a function of *x* showed the existence of several phases corresponding to superstructures of the TiSe_2_ reference cell, as a result of an ordering of iron and vacancies in the van der Waals gap for values of *x* > 0.2.^13,15^ It was found that hybridization of Ti 3d and Fe 3d states, along with the overlap of titanium and iron d_*z*^2^_ electron shells along the *c* axis leads to covalent bonding between layers; therefore a lattice compression occurs along the *c* axis, although the unit cell volume increases linearly with iron concentration.^16–18^

The Fe_*x*_TiSe_2_ system presents retrograde solubility in the solid state; an increase in the temperature of the system results in iron release, and on further heating it re-enters the lattice. This phenomenon is caused by the thermal expansion of the impurity band of the Fe 3d/Ti 3d hybrid states. Thermally induced phase transitions are of first order, and therefore the specimen state at the given temperature can be fixed by quenching.^18^

Bulk TiSe_2_ shows metallic conductivity and an optical band gap of 0.1 eV.^19^ Upon intercalation of high spin Fe^2+^ in the TiSe_2_ “host” structure, the electrons donated from iron contribute to the titanium t_2g_ band suppressing its charge density wave behaviour^20^ and increasing the resistivity of the material with increasing iron concentration.^11^ A transition from spin glass behaviour to an antiferromagnetic regime occurs at percolation threshold of intercalated iron *x* > 0.2 due to coupling between the iron magnetic moments and superexchange interactions.^13,17^

Conventional methods for preparation of binary and ternary transition metal dichalcogenides involve heating of high purity elements at high temperatures for long periods of time, and requires several homogenizing steps, making their synthesis long and expensive.^16,18,21^ The decomposition of metal chalcogenolato complexes to metal chalcogenides takes place at significantly lower temperatures, and they provide high purity materials required for electronic applications. Recent research has highlighted the importance of careful precursor design, in order to facilitate cleaner decomposition,^22^ lowering contamination and improving performance of the resultant functional material. Chemical vapour deposition (CVD) has drawn attention as a method to synthesis thin films of functional materials,^23^ including metal selenides.^24,25^ The use of single source precursors in CVD has a number of advantages, not least the simplification of the decomposition mechanism since all required elements are delivered to the desired substrate at the same time. Earlier this year compounds of the type: [Fe(η^5^-C_5_H_4_Se)_2_M(η^5^-C_5_H_5_)_2_]_2_ [M = Ti (1), Zr (2), Hf (3)] have been reported^22^ for potential utilization as single source precursors. In particular, the use of the titanium species 1 in the LPCVD fabrication of iron intercalated titanium diselenide Fe_*x*_TiSe_2_ could remove any pre-reaction issues or formation of unwanted side products and facilitates a facile, one step route to functional semiconductor materials with tuneable band gap.

There are only a handful of reports using CVD to produce thin films of metal chalcogenides.^19,26–29^ The work presented herein explores the capability of our precursors to generate high quality thin films of iron-doped titanium selenide, with the objective to achieve desired optical and electrical properties. Following the synthesis of the single source precursor, [Fe(η^5^-C_5_H_4_Se)_2_Ti(η^5^-C_5_H_5_)_2_] (1), LPCVD has been used to produce functional thin films of Fe_*x*_TiSe_2_. Here, we report for the first time, to the best of our knowledge, the simultaneous synthesis of polycrystalline powder Fe_0.46_TiSe_2_ and its thin film deposition. This approach could offer a faster alternative to the conventional synthetic route of iron-intercalated titanium diselenides, involving only a one-step heat treatment.

## Experimental section

### Precursor synthesis

The precursors were synthesized according to literature.^22^*N*,*N*,*N*′,*N*′-Tetramethylethylenediamine (Aldrich) was distilled over sodium and stored over sieves 3 Å (20% m/v) for 24 h and ferrocene (Merck, 99%) was freeze dried for 12 h prior to use. Selenium (shot, Aldrich), nBuLi (2.5 M in Hexane, Aldrich), ^*t*^BuLi (1.7 M in Hexane, Aldrich) and bis(cyclopentadienyl)titanium(iv)dichloride (Aldrich) were used as purchased. Dry THF (99.9% in Argon, Sigma) and dry toluene were stored over a sodium mirror for 24 h prior to use, and pre-dried dichloromethane was dried over Mo sieves 3 Å (20% m/v) for 48 h prior to use. All preparations were undertaken using Schlenk line techniques, and all glassware was dried for 12 h at 200 °C prior to use. Synthesis of the precursor was performed under argon, which was passed over a drying column. After isolation, the polycrystalline powder precursor was stored in a glovebox under an Argon atmosphere. Synthesis and purification of the precursor was confirmed by NMR:^22^^1^H NMR (600 MHz) *δ*/ppm (C_6_D_6_): 5.67 (s, 10H); 4.35 (m, 8H). ^13^C{^1^H} NMR (600 MHz) *δ*/ppm (C_6_D_6_): 110.99 (m, Fc); 111.26 (m, Cp).

### Deposition studies – apparatus and characterization

LPCVD experiments were carried out in a quartz tube under dynamic vacuum (10^−1^ torr) embedded inside a furnace to allow uniform heating. The temperature was controlled using Pt–Rh thermocouples. The polycrystalline precursor was spread evenly in a glazed ceramic boat (0.9 × 1.4 × 10.3 cm, VWR® Cat. no. 459–0224) and heated up to 1000 °C for 1–18 h in order to achieve its sublimation and the formation of the final product. Powders were collected alongside films on quartz slides (2.5 cm × 1.0 cm × 2 mm) supplied by Multi-Lab, which were cleaned using acetone (99%), isopropanol (99%), and distilled water and dried at 200 °C overnight prior to use. The precursor (*ca*. 0.15 g) sublimed and deposited over the slides in the hot zone of the reactor. After several attempts two cooling protocols were established: 1 – cooled at 13° min^−1^ to 450 °C and then quenched; 2 – gradual cooling of 13° min^−1^ to 355 °C, followed by 2 °C min^−1^ to room temperature. The tube was then transported inside the glovebox, where the remaining powder and the quartz slides were stored for characterization. The powder samples were grinded in a metal mortar inside the glovebox, loaded to 3 mm borosilicate capillaries and sealed for characterization.

PXRD data were collected on a STOE diffractometer using monochromated Mo K_α1_ radiation (0.70903 Å; 50 kV, 30 mA) and 4 scans per measurement over the range 2*θ* of 10–40°, with a step size of 0.5° and a count time of 10 s per step. GIXRD analysis was performed using a Bruker-Axs D8 (Lynxeye XE) diffractometer with monochromated Cu K_α1_ radiation (1.54184 Å; 20 kV, 5 mA). The films were analyzed with a grazing incident angle (*θ*) of 1°. Thin film XRD studies showed high fluorescence due to use of copper radiation and the presence of iron (Fig. S1†). Polycrystalline powders from the same experiments were therefore loaded into capillaries and analyzed using a STOE Stadi P diffractometer (Mo K_α1_ radiation, 0.70903 Å, 50 kV, 30 mA), in which less fluorescence was detected. For the thin films X-ray photoelectron spectroscopy (XPS) was performed using a Thermo K alpha spectrometer with monochromated Al Kα radiation (8.3418 Å), a dual beam charge compensation system and a constant pass energy of 50 eV. Survey scans were collected in the range of 0–1200 eV. High resolution peaks were used for the principal peaks of Ti (2p), Fe (2p), Se (3d), and C (1s). The peaks were modelled using sensitivity factors to calculate the film composition. The area underneath these bands was an indicator of the element concentration within the region of analysis (spot size 400 μm). Scanning electron microscope (SEM) studies were carried out using a JEOL 6301 (10 kV) and a JEOL JSM-6700F field emission instruments, after sputtering of the samples with a thin layer of gold for increased imaging. UV-vis spectroscopy was performed using a Shimadzu UV-2600 240 V IVDD UV/vis Spectrophotometer in the 350–900 nm range. A Labsphere reflectance standard was used as reference in the UV-vis measurements.

## Results and discussion

**Scheme 1:**
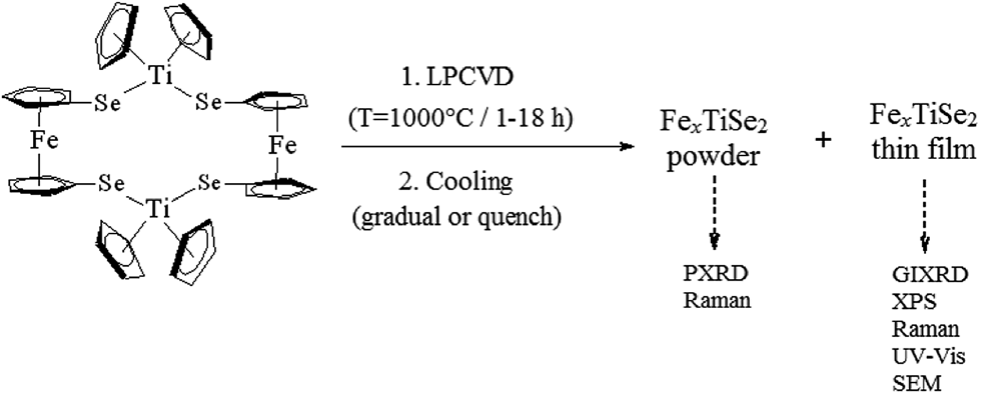
Scheme of the LPCVD of precursor (1) and analysis techniques for products (powders and films).

[Fe(η^5^-C_5_H_4_Se)_2_Ti(η^5^-C_5_H_5_)_2_]_2_ (1) was selected as a single source precursor, since the Fe doping is expected to tune the optical and electrical band gap of TiSe_2_. The Fe : Ti : Se ratio in 1 is 1 : 1 : 2, which facilitates an excess of iron dopant and therefore the maximum amount of intercalation into the targeted TiSe_2_ lattice. Following the synthesis and characterisation of 1,^22^ LPCVD studies produced thin films which were uniform, adherent and showed a good coverage of the substrate. For each experiment powder deposits were also collected and analysed using PXRD to calculate the composition using the Rietveld refinement (Scheme 1). These depositions on quartz substrates were conducted alongside ceramic boats containing 1 at 1000 °C, by varying the reaction time between one and eighteen hours. It was established that two different cooling processes were required in order to prove the retrograde solubility particular to the Fe/Fe_*x*_TiSe_2_ system, and find the best conditions for maximum amount of intercalated iron in the structure. In the first process the reactor was cooled to 13° min^−1^ until 450 °C and the quartz tube was subsequently quenched with water; in the second process a gradual cooling of the reactor was carried out at 13° min^−1^ until reaching 355 °C, followed by a slower cooling process of 2° min^−1^ until it reached room temperature. Subsequently, air and moisture sensitive black powder samples with a metallic shine, as well as black thin films on quartz (also exhibiting a metallic lustre) were handled under an inert environment for analysis.

This custom experimental setup of running the LPCVD and pyrolyzing the powder concurrently facilitated analysis, allowing higher quality XRD data to be collected from the powder samples.

**Fig. 1:**
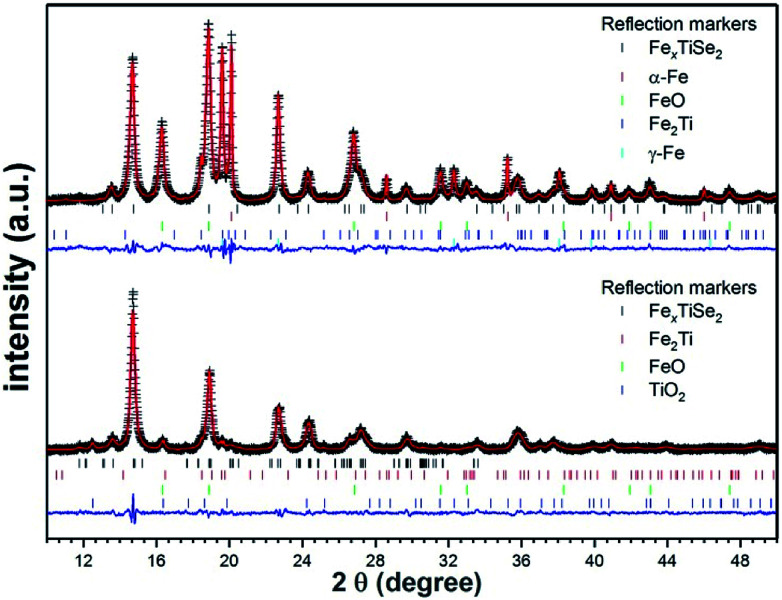
Rietveld refinement plots of the diffraction data collected from pyrolysis of 1 for 18 h followed by quenching (top) and gradual cooling (bottom). The crosses represent experimental diffraction pattern, the red line is the calculated pattern, and the blue line represents the difference *y*_obs_ − *y*_calc_. The calculated Bragg angles (2*θ*) are marked by the vertical bars. Structural analysis was performed using the GSAS package.^30^ For clarity, the displayed data are background-subtracted to remove the large contribution from Fe fluorescence.

**Fig. 2:**
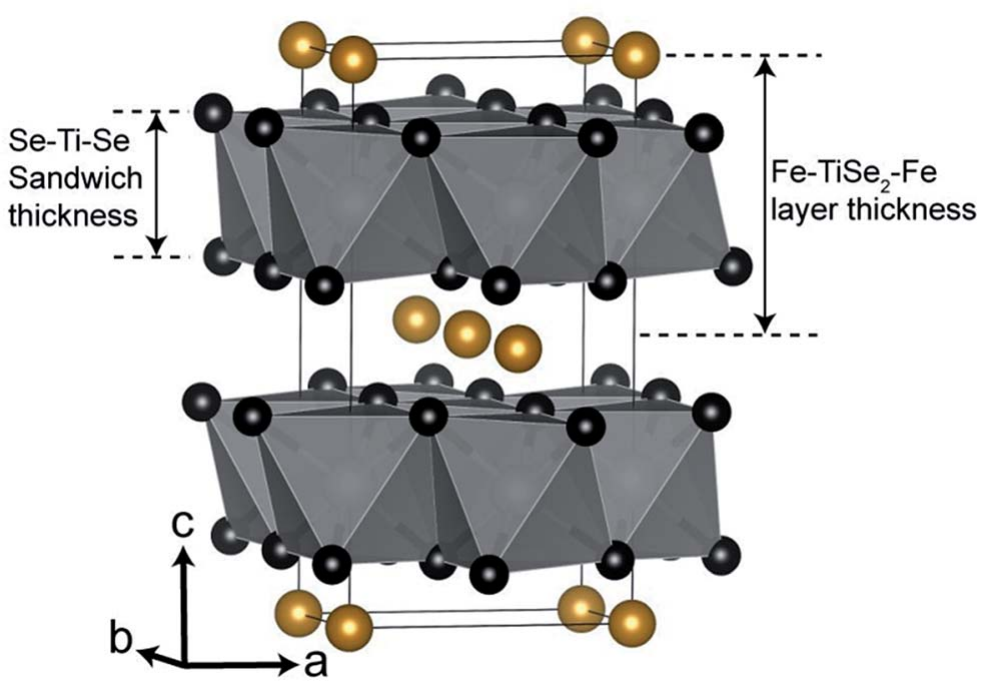
Structural diagram of Fe_0.48_TiSe_2_. Titanium atoms in white (inside octahedra), selenium atoms in black and iron atoms in gold.^41^

The thin films deposited *via* LPCVD were analysed using GIXRD, however as a result of the Cu K_α1_ radiation used, fluorescence effects due to the content of iron in the films made characterisation unreliable (Fig. S1†). Polycrystalline powders of LPCVD products were examined by PXRD using Mo K_α1_ source in order to reduce the fluorescence effects in the patterns. The PXRD patterns of the powder samples produced confirmed the formation of the intercalation compounds Fe_*x*_TiSe_2_ (Fig. 1). Structural refinement using Rietveld analysis of the data confirmed the maximum intercalation of iron where *x* = 0.48(2) (Table 1), which structure is shown in Fig. 2.

**Table 1:** Refined parameters and fit indicators for the Rietveld refinement of diffraction data for pyrolysis of 1 for the two different cooling protocols

Cooling protocol	Gradual	Quenched
Fe_*x*_TiSe_2_ space-group	*I*2/*m*	*P*3̄*m*1
Lattice parameters (Å, °)	*a* = 6.2040(13), *b* = 3.6070(3), *c* = 11.9255(16), *β* = 89.69(2)	*a* = 3.5994(2), *c* = 5.9932(6)
Fe fractional occupancy, *x*	0.48(2)	0.254(14)
Se–Ti–Se sandwich thickness (Å)	3.126(7)	3.009(12)
Fe_*x*_TiSe_2_ phase fraction (wt%)	82.4(8)	29.75(12)
Impurity phases and fractions (wt%)	Fe_2_Ti = 1.35(8), FeO = 6.6(3), TiO_2_ = 9.7(4)	α-Fe = 6.9(4), γ-Fe = 17.3(6), FeO = 35.2(9), Fe_2_Ti = 10.8(2)
Goodness of fit, w*R*_p_, *R*_p_, *χ*^2^	1.37, 1.02, 2.139	1.26, 0.96, 3.88

As can be seen in the PXRD data (Fig. 1), the two cooling protocols lead to distinctly different products subsequent to identical pyrolysis. The observation of substantial elemental and oxidised iron in the quenched sample suggests a lower level of intercalation within the TiSe_2_ structure, whilst the slow-cool protocol leads to a significantly reduced level of un-intercalated Fe. A distortion from the hexagonal *P*3̄*m*1 space-group of the parent TiSe_2_ compound has previously been observed upon intercalation of Fe at levels greater than *x* = 0.2.^16^ At a value of *x* = 0.25 and *x* = 0.5, superstructures are observed due Fe and vacancy ordering as well as a monoclinic distortion.^15^

Whilst the Fe_*x*_TiSe_2_ phase seen in the quench-cooled material is best described using the parent hexagonal *P*3̄*m*1 space-group, the fit of the slow-cooled Fe_*x*_TiSe_2_ structure is significantly improved when using the distorted *I*2/*m* space-group. Intercalation of Fe between layers results in a covalent Fe–Ti–Fe bond and has been shown to decrease the inter-layer spacing and subsequently the *c* lattice parameter. Ignoring the small monoclinic distortion (*β* = 89.69(2)°) the *I*2/*m* structure is related to the parent *P*3̄*m*1 structure through the conversion *a* = *a*′√3, *b* = *a*′, *c* = 2*c*′. An increase in the total layer width, Fe–TiSe_2_–Fe (the Se–Ti–Se sandwich thickness plus the Fe-containing van-der-Waals gap Se–Fe–Se), when comparing the quench cooled protocol (*c* = 5.9932(6) Å) and the slow cooled (*c*/2 = 5.9629(7) Å) are in line with those seen in previous studies and confirm the reduced Fe content. Shkvarina *et al.* also found that whilst the total layer (Fe–TiSe_2_–Fe) width decreases on Fe-doping, an increase in the Se–Ti–Se sandwich thickness is observed due to weakening of the Ti–Se bonding.^18^ We do observe a slight increase in Se–Ti–Se thickness in refinements of the slow-cooled, higher Fe-doped, material (3.126(7) *vs.* 3.009(12) Å) but the values are almost within error and higher quality data would be needed to confirm this feature.

The observed space-group symmetry, the refined Fe content and the inter-layer lattice parameter all confirm the increased intercalated-Fe content upon gradual cooling of the sample, compared to quench-cooling from 450 °C. This is further evidenced by the observation of large fractions of both elemental and oxidised Fe as impurities in the quench cooled sample. These observations are concordant with those reported for materials such as Fe_*x*_TiSe_2_ showing retrograde Fe solubility on heating.^18^ At temperatures >1000 °C a large *x* value is obtainable within the lattice but on cooling Fe becomes less soluble being seen instead as a mixed phases of Fe and Fe_*x*_TiSe_2_. At lower temperatures, solubility again increases and greater values of *x* are achievable.^18^ During precursor decomposition and Fe_*x*_TiSe_2_ deposition, a high intercalation level is expected. On cooling the film, Fe solubility changes and a mixture of discrete Fe and Fe_*x*_TiSe_2_ phases should then be observed. Quench-cooling from this mixed-phase regime is expected to lead to the observation of a significant elemental Fe content and a reduced level of intercalation, in line with our results. Gradual cooling, however, gives the lattice time to allow Fe-reintercalation, increasing *x*, and decreasing the level of observed elemental or subsequently oxidised iron.

**Fig. 3:**
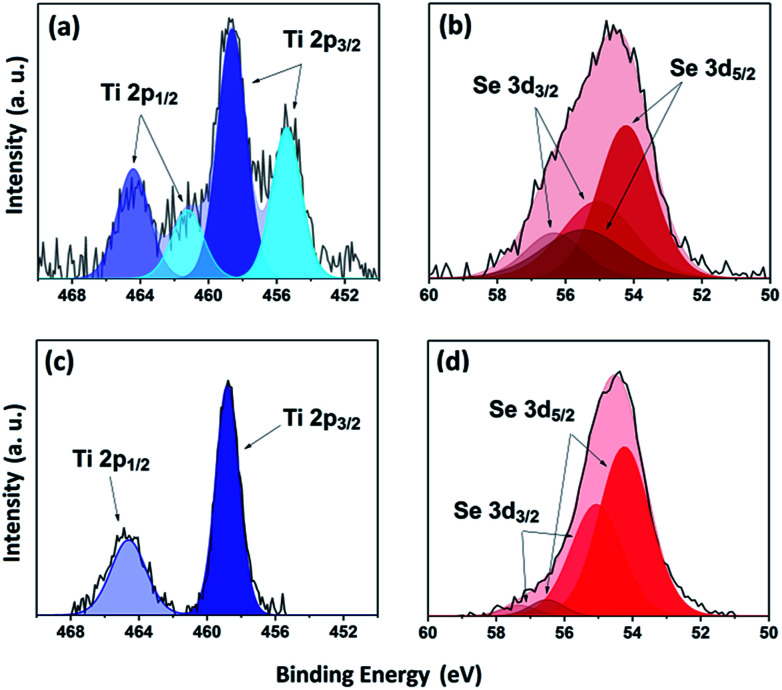
XPS transitions for powder (top) and film surface (bottom) of Ti 2p (a and c) and Se 3d (b and d) environments of Fe_0.48_TiSe_2_.

XPS was used to study the environment of the gradually cooled Fe_0.48_TiSe_2_ powder sample and thin films. As XPS is a surface-sensitive technique (≤10 nm),^31^ several depths within the bulk of the film were investigated by etching the surface with argon sputtering. Previous works have shown that TiSe_2_ materials present a Ti2p environment at 456.2 eV (ref. 32) whereas for Fe_0.40_TiSe_2_ materials the Ti 2p_3/2_ band appears at 455.2 eV.^33^ XPS measurements of the powder sample exhibit two titanium environments with Ti 2p_3/2_ = 455.4 and 458.7 eV, corresponding to Ti^4+^ species of respectively TiSe_2_ (ref. 32) and TiO_2_, respectively^34^ (Fig. 3a). Additionally two selenium environments were observed, the first: 3d_5/2_ = 54.2 eV, 3d_3/2_ = 55.0 eV corresponds to that of TiSe_2_ (Se 3d_5/2_ = 54.1 eV)^32^ while the second: 3d_5/2_ = 55.4 eV, 3d_3/2_ = 56.3 eV coincides with Se^0^ (Fig. 3b), likely due to decomposition of the sample during exposure to air prior to measurement.^32,35^

The thin films showed a single environment with components at Ti 2p_3/2_ = 458.7 eV and Ti 2p_1/2_ = 464.6 eV at the surface, demonstrating the formation of superficial TiO_2_ (ref. 34) due to the exposure of the film to air prior to its measurement (Fig. 3c). It should be noted that the process of sputtering under argon while etching reduces the Ti^4+^ species to Ti^3+^ and cannot be easily fitted. For the Fe_0.48_TiSe_2_ thin film, two components of a major environment Se 3d_5/2_ and 3d_3/2_ were observed at 54.2 and 55.1 eV (Fig. 3d), consistent with the existence of Se^2−^ species of TiSe_2_.^32^ It is worth noting that a minor second environment in the thin film was observed for Se at 56.5 eV (3d_5/2_) and 57.3 eV (3d_3/2_). It could be attributed to Se–Fe–Se interaction, further evidencing the intercalation of iron (Fig. 3d).^35,36^ XPS profile of selenium remains the same upon etching, therefore no other environments of selenium are detected in the film.

Owing to the well documented multiplet splitting in species of high spin Fe(ii), XPS spectra cannot be fitted quantitatively for samples containing Fe_*x*_TiSe_2_.^37^ Nonetheless, a clear visual change in the Fe XPS of the samples is apparent (as can be evidenced in Fig. S2†). It is highly likely that Fe_3_O_4_ is present both in the powder and in the surface of the thin film due to exposure to air. However, these species were not found upon XPS depth profile analysis in the thin film. As a result of the monoclinic structural distortion in Fe_0.48_TiSe_2_, an ordering of iron atoms results in the formation of a sequence of octahedral site chains in the van der Waals gaps.^33^ XPS of inner layers (300 s etch) showed a Fe 2p environment at 706.7 eV and 719.2 eV (Fig. S2†), which coincides with the band corresponding to the metallic Fe–Fe bond in Fe_0.40_TiSe_2_.^33^ Interestingly, previous works have reported that FeSe_2_ materials present a Fe 2p environment at 2p_3/2_ = 707.1 eV and 2p_1/2_ = 719.8 eV, which would corroborate the presence of Fe^2+^ species intercalated between layers of selenium.^35^

Carbon contamination was found in the surface, which decreased upon etching (Fig. S3†). Elemental ratios could not be further corroborated by XPS analysis due to the air sensitivity of the samples.

**Fig. 4:**
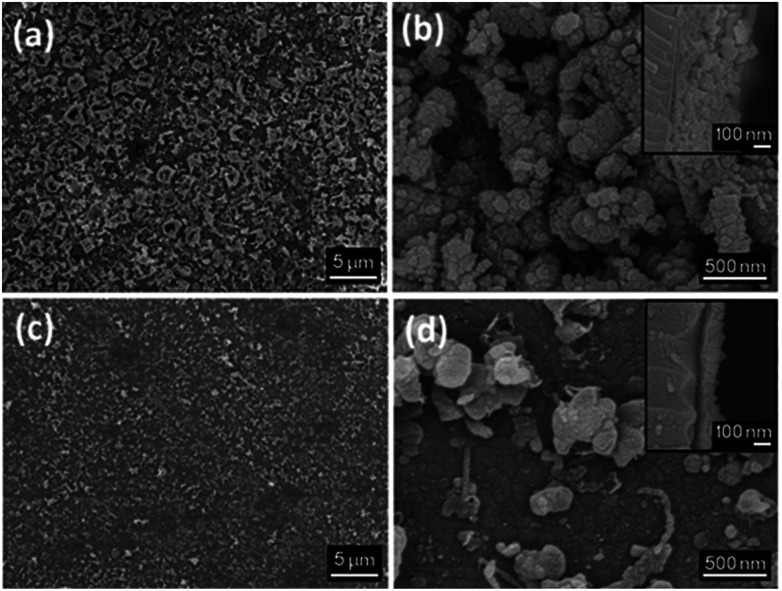
SEM micrographs of Fe_0.48_TiSe_2_ deposited at 1000 °C for 1 h (a and b) and for 18 h (c and d). Augmentation for micrographs are ×3000 for (a) and (c), and ×40 000 for (b) and (d). Side-on SEM micrographs of each sample are shown in the top right corners.

SEM images of the thin films display growth of clusters of crystallites, resulting in a “bubbled” surface (Fig. 4a and b). The films deposited for 1 h display an irregular morphology, promoting the absorption of moisture as well as preventing a smooth deposition of a potential coating material. The effect of annealing under a static vacuum for 18 h leads to a smoother surface and an decrease in film thickness (Fig. 4c and d). A compacting process of the film occurs upon annealing, resulting into a decrease if film thickness. Film thicknesses are ∼250 nm for the film deposited for 1 h and ∼120 nm for the film deposited for 18 h (annealed).

**Fig. 5:**
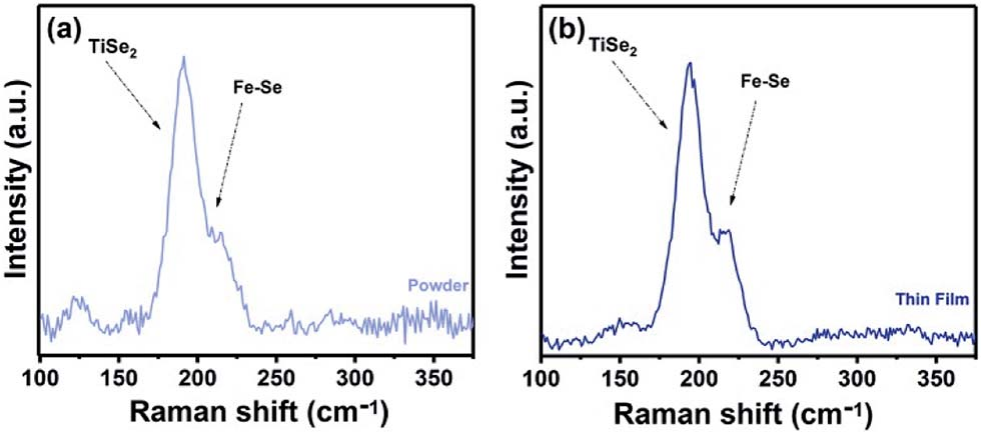
Raman spectra of (a) powder and (b) thin film sample of Fe_0.48_TiSe_2_ produced at 1000 °C for 18 h using the gradual cooling protocol.

**Fig. 6:**
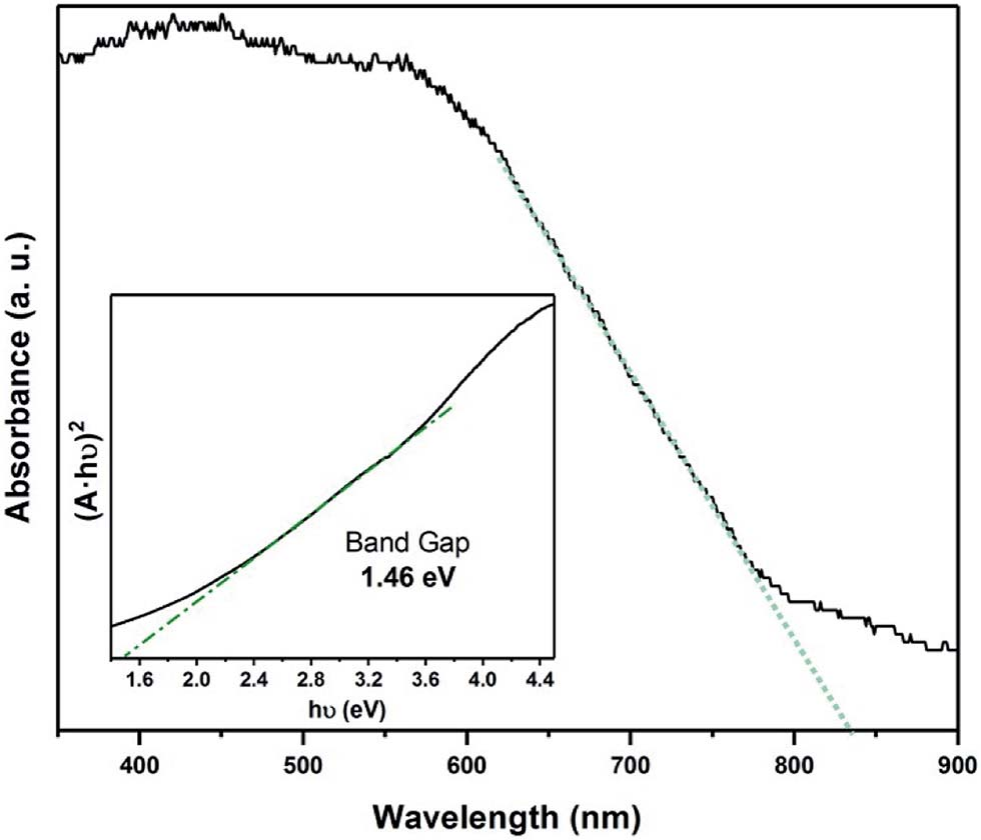
Optical absorption spectrum of Fe_0.48_TiSe_2_ calculated from transmittance (*A* = 2 − log_10_ *T*%) and band gap calculation using a Tauc plot.

Raman spectroscopy was used to study of the powder samples and the Fe_*x*_TiSe_2_ thin films. Raman spectra of both powders and thin film corresponding to the gradual cooling process exhibit a characteristic band at 195 cm^−1^, very close to the A_1g_ band of TiSe_2_,^38^ as well as a band at 218 cm^−1^ in the area expected for a Fe–Se band (Fig. 5).^39^ The Raman spectroscopic study of the “quenched” film revealed the characteristic strong bands for Fe_3_O_4_, as a result of the rapid oxidation of the excess iron (Fig. S4†).^40^ The optical absorption spectrum Fe_0.48_TiSe_2_ was calculated from transmittance measurements of the films, and it is shown in Fig. 6. The variation of (*αhν*)^2^*versus* the *hν* was linear at the absorption edge, which confirmed that Fe_0.48_TiSe_2_ is a semiconductor with a direct band gap of 1.46 eV (Fig. 6). As such these new Fe-doped TiSe_2_ films are a breakthrough in the development of multifunctional advanced materials with tuneable properties for a wide range of applications.

## Conclusions

Simultaneous synthesis of iron-intercalated TiSe_2_ powder samples and thin films from the single source precursor 1 were achieved by treatment at 1000 °C for periods of 1–18 h. The use of two distinct cooling protocols yielded Fe_*x*_TiSe_2_ materials with different degree of iron intercalation. Maximum intercalation was reported with the formation of Fe_0.48_TiSe_2_, performing a gradual cooling process to room temperature. Raman spectroscopy for this sample confirmed both thin films and powders synthesized in the same experiment to be the same material. Intercalation of iron with a fractional occupancy of 0.48 ± 2 in the host structure of layered TiSe_2_ shows a significant increase in the band gap from 0.1 eV to 1.46 eV, which lies close to the limit of maximum solar conversion efficiency (Shockley–Queisser limit) and is consistent with potential application as p-type absorber layer in photovoltaic cells. The development of thin film technology has revolutionized our way to design materials with specific structures and to integrate these architectures into functional devices. For the first time, thin films of iron-doped titanium diselenide have been deposited through a convenient one-step heat process, opening a range of potential applications in the field of optoelectronics and the solar energy industry.

## Conflicts of interest

There are no conflicts to declare.

## Supplementary Material

RA-008-C8RA03174F-s001
