# Supplementary material for: Accessing new 2D semiconductors with optical band gap: synthesis of iron-intercalated titanium diselenide thin films via LPCVD
Source: RSC Adv. 2018 Jun 20;8(40):22552–8. doi: 10.1039/c8ra03174f (PMC9081417; doi:10.1039/c8ra03174f)
Supplement: RA-008-C8RA03174F-s001 [file RA-008-C8RA03174F-s001.pdf]

Electronic Supplementary Material (ESI) for RSC Advances.
This journal is © The Royal Society of Chemistry 2018

Accessing new 2D semiconductors with optical band gap: Synthesis of Iron-intercalated Titanium Diselenide thin films via LPCVD.

Clara Sanchez-Perez,[a,b] Caroline E. Knapp,[a] Ross H. Colman,[c] Carlos Sotelo-Vazquez,[a] Raija Oilunkaniemi,[b] Risto S. Laitinen,*[b] Claire J. Carmalt*[a]

Phase	Space-group	*a* (Å)	*b* (Å)	*c* (Å)	*β* (°)	Phase fraction (wt. %)
	Atom	*x/a*	*y/b*	*z/c*	Occ	U_{iso} (Å^2)
Fe_xTiSe_2	*P-3m1*	3.5994(2)	3.5994(2)	5.9932(6)	90	29.75(12)
	Ti	0	0	0	1.0	0.012(3)
	Se	1/3	2/3	0.2511(14)	1.0	0.002(5)
	Fe	0	0	0.5	0.254(14)	0.011
α-Fe	*Im-3m*	2.8697(5)	2.8697(5)	2.8697(5)	90	6.9(4)
	Fe	0	0	0	1	0.002
γ-Fe	*Fm-3m*	3.60569(8)	3.60569(8)	3.60569(8)	90	17.3(6)
	Fe	0	0	0	1	0.007
FeO	*Fm-3m*	4.32290(13)	4.32290(13)	4.32290(13)	90	35.2(9)
	Fe	0	0	0	1	0.0017
	O	0.5	0.5	0.5	1	0.0097
Fe_2Ti	*P63/mmc*	4.7967(13)	4.7967(13)	7.832(2)	90	10.8(2)
	Fe	0	0	0	1	0.085
	Fe	0.830	0.659	1/4	1	0.388
	Ti	1/3	2/3	0.065	1	0.135

Table S1. Rietveld refined parameters from fitting the PXRD pattern of the *Quenched* sample, using GSAS and EXPGUI.[1] The background was modelled using a *Shifted Chebyschev* function (GSAS background function 1) with 10 parameters. All profiles were modelled using a pseudo-Voigt peak shape (GSAS CW profile function 2). The quality of the data was not sufficient for refinement of impurity phase structural details and so only lattice and profile parameters were allowed to refine. The thermal parameter of the Fe_xTiSe_2 intercalated iron was also found to be unstable in the refinement and so was fixed at a physically reasonable value. Goodness of fit parameters are reported in the main text.

Whilst the appearance of γ(FCC)-Fe at room-temperature as an impurity is initially unexpected, it has previously been stabilised and extensively studied as epitaxially grown films on Cu(001) and diamond surfaces.[2,3,4] The FCC structure is stabilised by lattice matching the substrate (e.g. FCC-Fe *a* ~ 3.59 Å, Cu(001) *a* = 3.61 Å, diamond *a* = 3.57 Å) and so it's appearance on Fe_xTiSe_2 (*a* = 3.60 Å) is reasonable. An alternative, although slightly less likely, explanation for the observation is the possible growth of FCC-Fe filled carbon-nanotubes. These structures have been observed through decomposition of ferrocene at high temperatures, similar to our film growth conditions and CVD precursor.[5,6]

Phase	Space-group	*a* (Å)	*b* (Å)	*c* (Å)	*β* (°)	Phase fraction (wt. %)

	Atom	*x/a*	*y/b*	*z/c*	Occ	U_{iso} (Å^2)
Fe_xTiSe_2	*I2/m*	6.2040(13)	3.6070(3)	11.9255(16)	89.69(2)	82.4(8)
	Ti	-0.013(2)	0	0.2437(8)	1.0	0.008(2)
	Se1	0.1452(9)	0.5	-0.1240(4)	1.0	0.0022(4)
	Se2	0.3299(11)	0	0.1189(4)	1.0	0.0022(4)
	Fe	0	0	0	0.48(2)	0.0029
Fe_2Ti	*P63/mmc*	4.973(2)	4.973(2)	7.722(4)	90	1.35(8)
	Ti	1/3	2/3	0.065	1	0.013
	Fe	0	0	0	1	0.013
	Fe	0.83	0.659	1/4	1	0.013
FeO	*Fm-3m*	4.3250(14)	4.3250(14)	4.3250(14)	90	6.6(3)
	Fe	0	0	0	1	0.013
	O	1/2	1/2	1/2	1	0.013
TiO_2	*P42/mnm*	4.603(3)	4.603(3)	2.950(3)	90	9.7(4)
	Ti	0	0	0	1	0.013
	O	0.305	0.305	0	1	0.013

Table S2. Rietveld refined parameters from fitting the PXRD pattern of the *Gradual* cooled sample, using GSAS and EXPGUI. The background was again modelled using a *Shifted Chebyschev* with 10 parameters. All profiles were modelled using a pseudo-Voigt peak shape (GSAS CW profile function 2). The quality of the data was not sufficient for refinement of impurity phase structural details and so only lattice and profile parameters were allowed to refine. The thermal parameter of the Fe_xTiSe_2 selenium atoms were constrained to be equal. Goodness of fit parameters are reported in the main text.

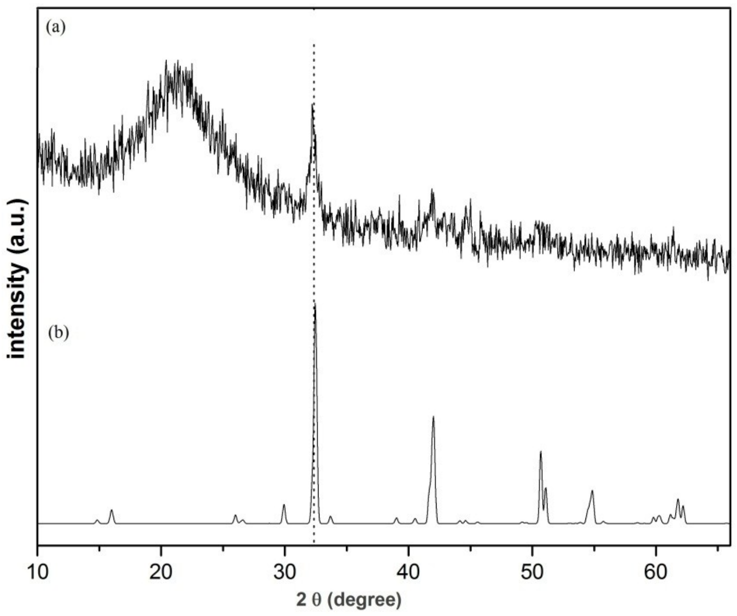

Figure S1. PXRD pattern of a $Fe_{0.48}TiSe_2$ film (a) prepared by LPCVD of 1 at 1000 °C for 18h, followed by gradual cooling to room temperature. (b) XRD standard pattern for the $Fe_{0.48}TiSe_2$ material, extracted from ICSD.[7] The pattern shows high fluorescence of iron with the copper beam from the anode on the X-Ray instrument.

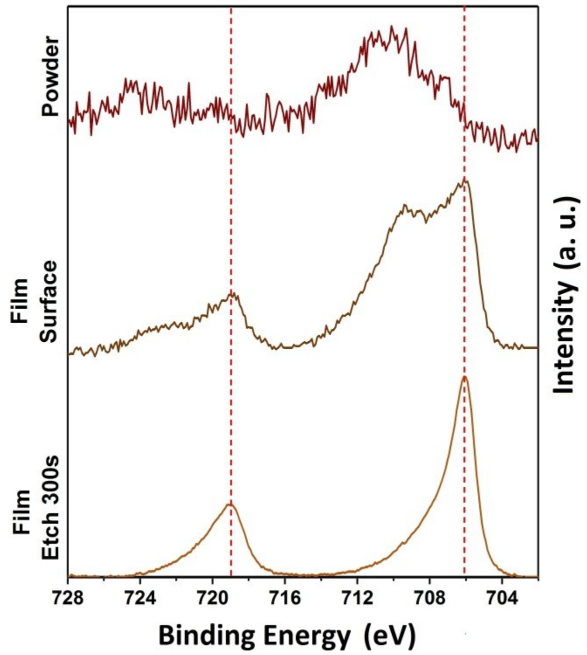

Figure S2. A visual change in the Fe XPS of the samples is apparent. It is highly likely that Fe_3O_4 species is present both in the powder and in the surface of the thin film due to its exposure to air (main peak at BE = 710 eV). Upon XPS depth profile analysis in the thin film, inner layers (300 s etch) showed a Fe 2p environment at 706.7 eV and 719.2 eV.

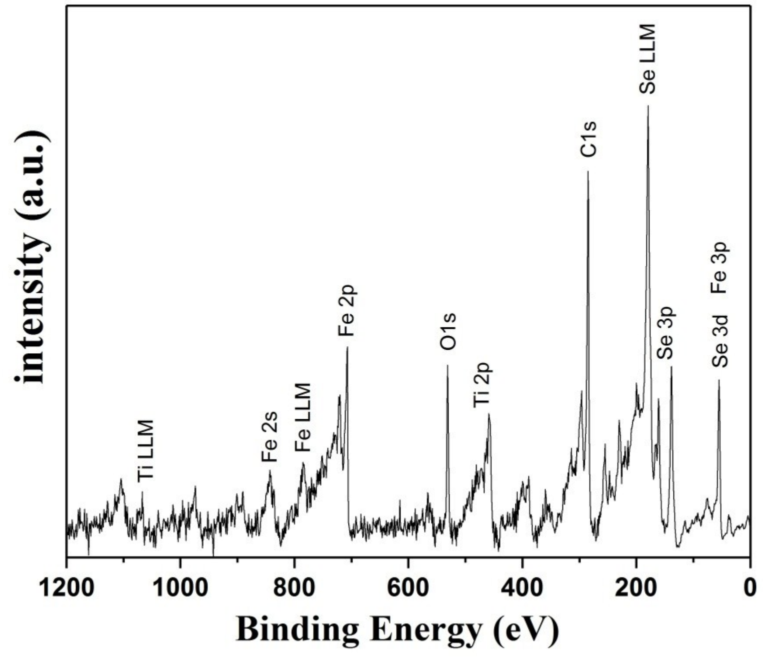

Figure S3. XPS survey scan of $Fe_{0.48}TiSe_2$ sample (0 - 1200 eV).

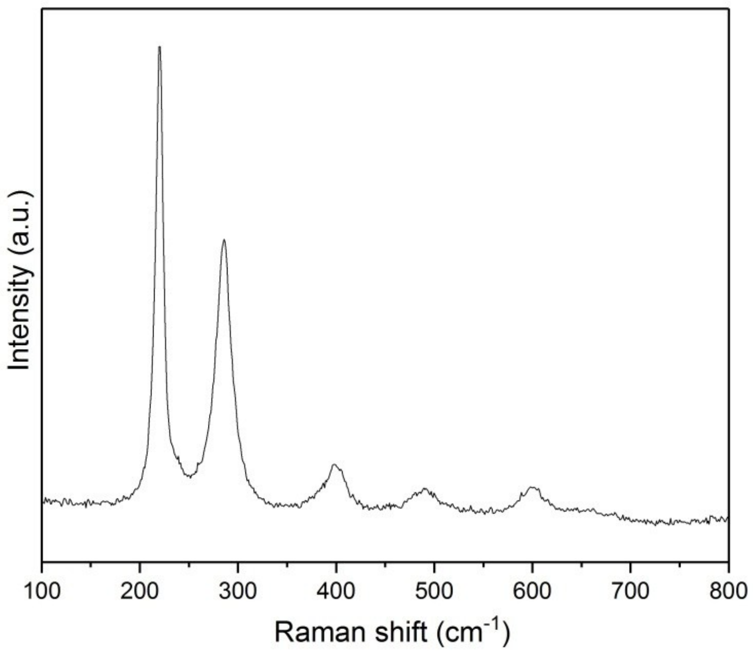

Figure S4. Strong bands for Fe_3O_4[8] detected using Raman spectroscopy of the film synthesized *via* “quenching” at 450 °C.

1. Toby, B. H. *et al. EXPGUI* , a graphical user interface for *GSAS*. *J. Appl. Crystallogr.* **34,** 210–213 (2001).
2. Related, P. S. Magnetism and Anisotropy of Ultrathin Epitaxial Austenitic Fe-Films. 3–6 (1989).
3. Magnan, H., Chandesris, D., Villette, B., Heckmann, O. & Lecante, J. Structure of thin metastable epitaxial Fe films on Cu(100): Reconstruction and interface ordering by coating. *Phys. Rev. Lett.* **67,** 859–862 (1991).
4. Pappas, D. P. *et al.* Growth of fcc Fe films on diamond. *Appl. Phys. Lett.* **64,** 28–30 (1994).
5. Kim, H., Kaufman, M. J., Sigmund, W. M., Jacques, D. & Andrews, R. Observation and formation mechanism of stable face-centered-cubic Fe nanorods in carbon nanotubes. *J. Mater. Res.* **18,** 1104–1108 (2003).
6. Wei, B. *et al.* Room-temperature ferromagnetism in doped face-centered cubic Fe nanoparticles. *Small* **2,** 804–809 (2006).
7. Calvarin, G., Gavarri, J. R., Buhannic, M. A., Colombet, P. & Danot, M. Crystal and magnetic structures of Fe0.25TiSe2 and Fe0.48TiSe2. *Rev. Phys. Appliquée* **22,** 1131–1138 (1987).
8. Song, K., Lee, Y., Jo, M. R., Nam, K. M. & Kang, Y.-M. Comprehensive design of carbon-encapsulated Fe $_3$ O $_4$ nanocrystals and their lithium storage properties. *Nanotechnology* **23,** 505401 (2012).
